# This was the year that was: brain barriers and brain fluid research in 2019

**DOI:** 10.1186/s12987-020-00181-9

**Published:** 2020-03-05

**Authors:** Richard F. Keep, Hazel C. Jones, Lester R. Drewes

**Affiliations:** 1 Department of Neurosurgery, University of Michigan, R5018 BSRB, 109 Zina Pitcher Place, Ann Arbor, MI 48109-2200 USA; 2 Gagle Brook House, Chesterton, Bicester, OX26 1UF UK; 3 Department of Biomedical Sciences, University of Minnesota Medical School Duluth, Duluth, MN 55812 USA

## Abstract

This editorial highlights advances in brain barrier and brain fluid research published in 2019, as well as addressing current controversies and pressing needs. Topics include recent advances related to: the cerebral endothelium and the neurovascular unit; the choroid plexus, arachnoid membrane; cerebrospinal fluid and the glymphatic hypothesis; the impact of disease states on brain barriers and brain fluids; drug delivery to the brain; and translation of preclinical data to the clinic. This editorial also mourns the loss of two important figures in the field, Malcolm B. Segal and Edward G. Stopa.

## Editorial

Brain barrier and brain fluid research is thriving. For example, an Ovid MEDLINE search of the terms, “blood–brain barrier”, “brain + endothelium”, “neurovascular unit”, “cerebrospinal fluid”, “choroid plexus”, “arachnoid membrane”, “hydrocephalus”, “brain edema”, or “glymphatic system” lists ~ 9300 papers for 2019. This editorial highlights some of those papers and underlying themes for the *Fluids and Barriers of the CNS* readership.

The papers cited reflect the interests of the Editors-in-Chief and the list is not intended to be exhaustive. We touch on topics to, hopefully, inspire readers to a greater exploration of particular areas. As always, we welcome submission of more in-depth reviews. In 2019, we had a thematic series on, *CNS Fluid and Solute Movement: Physiology, Modelling and Imaging*. In 2020, we will have a thematic series on, *Advances in In Vitro Modeling of the Blood*–*Brain Barrier and Neurovascular Unit.*

## Tools

In the past few years, one major advance has been the introduction of human induced pluripotent stem cells (iPSCs) to produce different cell types composing the neurovascular unit (NVU). Methods have now been extended to produce not only iPSC-derived endothelial cells, astrocytes and neurons, but also pericyte-like cells [1]. Improved methods for producing NVU cells are continuously being developed (e.g. [2]) and all four cell types can be produced and co-cultured from the same iPSC donor [3]. Currently, studies are examining the effects of individual patient mutations using iPSC-derived endothelial cells [4]. The ability to derive multiple cell types from a single donor enables examination of not only the effects of mutations on a single cell type but also on the complete NVU (e.g. on cell to cell communication). One use of human iPSCs has been to produce cerebral organoids that have allowed studies of human brain development and the complex interactions between different cell types. Importantly, such organoids can now incorporate a vasculature that is perfused and has BBB characteristics [5]. There continue to be advances in the use of iPSCs in engineering a BBB-on-a-chip [6–8].

Many recent advances in brain barrier and brain fluid research have been driven by improvements in imaging. Now, super-resolution microscopy techniques [9] have opened the possibility of using light microscopy to examine cells, including live cells, at much greater resolution and these methods can be applied to the cerebral endothelium. Examples are structured illumination microscopy (SIM) to examine the actin cytoskeleton [10], and stochastic optical reconstruction microscopy (STORM) for changes in endothelial junction organization [11] and glycocalyx molecular structure [12]. Imaging using magnetic resonance (MR) to quantify brain metabolites and to measure dynamic changes is expanding. This technology has been applied to the blood–brain transport of lactate in brain and may also be applicable to other metabolites [13].

One issue in NVU research is how to evaluate the role of different cell types. For example, Dieguez-Hurtado et al. [14] found that inactivating a transcription factor, RBPJ, post-natally alters pericyte function, causing vascular instability and lesions with some similarities to cerebral cavernous malformations, and greater injury after ischemic stroke. Another advance for manipulating the NVU and brain (experimentally and therapeutically) is the finding by Alterman et al. [15] that a single CSF injection of divalent small interfering RNAs is effective in reducing huntingtin expression (mRNA and protein) throughout the mouse brain, with effects lasting for at least 6 months. They had similar results in cynomolgus macaques.

Other tools of relevance to brain barriers and brain fluids research have been developed. Tang et al. [16] found that peptides containing a conserved motif (phenylalanine-arginine-tryptophan) bind to the cerebrovasculature but not to other vascular beds. The binding appears to be at the endothelial junctions but, interestingly, the peptides do not bind to the retinal vasculature. Understanding this phenomenon further may aid in the development of brain-targeting therapeutics. Another technique impacting biological and medical research is the use of machine learning. For example, in brain, machine learning can be used for image analysis [17, 18]. Machine learning is poised to impact brain barriers and brain fluids research in, for example, analysis of brain edema on radiographic images [19, 20] or potentially to predict brain drug penetration [21, 22].

## The cerebral endothelium and the neurovascular unit

### Crosstalk and cellular signaling within the NVU

We are gaining greater insight into endothelial-neuronal signaling. For example, Tan et al. [23] found that semaphorin 3G release from brain endothelial cells regulates synaptic structure and plasticity in the hippocampus. Similarly, Wu et al. [24] found that engrafting endothelial cells into brain promoted synaptogenesis and improved neurological deficits in neonatal hypoxia–ischemia in mice.

The past few years have demonstrated the importance of Wnt/β-catenin signaling in promoting a BBB phenotype. A recent example of the importance of oligodendroglial-vascular communication is in multiple sclerosis where abnormal perivascular clustering of oligodendrocyte precursor cells affects barrier function and inflammation by impacting Wnt signaling (Niu et al. [25]). Interestingly, two recent studies have indicated that low endothelial Wnt/β-catenin signaling underlies blood vessel leakiness in the circumventricular organs that lack a BBB [26, 27].

The critical role of another type of glial cell, the astrocyte, at the NVU including BBB regulation has long been known (reviewed in [28]). Recently, Bonsack et al. [29] demonstrated the importance of astrocyte-derived pentraxin-3 in regulating BBB permeability. Tan et al. [30] have also shown that leukocytes can cause BBB disruption in stroke via effects on astrocytes. Leukocytes release interleukin-9 that causes astrocytes to release vascular endothelial growth factor-A which, in turn, causes BBB hyperpermeability. S100B is also released from astrocytes during brain injury and it is now becoming clear that S100B is a Damage-Associated Molecular Pattern (DAMP) molecule that affects multiple cell types [31].

The importance of the pericyte at the NVU has become evident in recent years [32]. For example, Nikolakopoulou et al. [33] have now shown that pericyte ablation results in vascular dysfunction, including BBB breakdown and reduced blood flow, and neuronal loss. The latter is linked to loss of a pericyte-derived growth factor, pleiotrophin, that makes neurons vulnerable to ischemic injury. Another example is from Coucha et al. [34] who found that specifically targeting pericyte Ephrin-B2 can inhibit neovascularization in the brain of diabetic rats.

One question arising in multiple neurological conditions is how a sporadic mutation in just a few cells can result in a change in overall phenotype. Malinverno et al. [35], studying cerebral cavernous malformation-3 (CCM3), have found that CCM3 null brain endothelial cells clonally expand and then recruit other normal endothelial cells and induce them to express a mesenchymal/stem cell phenotype (endothelial cell: endothelial cell communication). One family of molecules involved in cell: cell communication is the connexins that form gap junctions. Recent evidence suggests gap junction activity may also regulate tight junction function at the BBB. Thus, in a mouse CCM3 model, Johnson et al. [11] found that brain endothelial cells overexpress connexin 43 enhancing not only gap junction communication, but also inducing tight junction disruption and BBB hyperpermeability.

While most studies have focused on proteins/peptides as mediators of signaling between cells of the NVU, there is considerable evidence on the importance of microRNAs in BBB regulation (reviewed in [36, 37]). There is also new evidence on the importance of another type of non-coding RNA, the long non-coding RNAs, at the BBB [37–39]. For example, some long non-coding RNAs play an important role in stroke by modulating mRNA and microRNA function [40].

Non-coding RNAs and mRNAs may be released from cells encapsulated (along with other components) in exosomes/extracellular vesicles. Such encapsulation increases microRNA stability and exosomal microRNAs have multiple effects in the CNS including at the cerebral endothelium [41, 42]. Exosome encapsulation is also proposed as one mechanism that might enable microRNAs to cross the BBB for therapy [41]. One element that has hampered exosome research at the cerebral endothelium is uncertainty over what receptors are responsible for their uptake. Now, Kuroda et al. [43] suggest that CD46 is an important receptor for exosomes derived from a brain-metastatic cell line.

The role of epigenetics in affecting brain function and neurological diseases has been the subject of much research. At the BBB, histone deacetylases (HDACs) have a role in regulating p-glycoprotein expression and activity [44]. Similarly, HDAC3 inhibition can reduce the endothelial disruption induced by oxygen and glucose deprivation (an in vitro ‘stroke’ model) [45] adding to data on the importance of epigenetic mechanisms at the NVU during stroke [37]. Other signaling molecules that have deacetylase activity are the sirtuins that are important in aging. An endothelial-specific mouse knockout of sirtuin-6 exacerbates BBB damage after cerebral ischemia [46] and Stamatovic et al. [47] found a decline in brain endothelial sirtuin-1 with normal aging in mouse and human that leads to BBB dysfunction. Recently, Senatorov et al. [48] reported the importance of BBB dysfunction with aging and how it can induce neural dysfunction through transforming growth factor-β signaling. In an interesting study, Yousef et al. [49] found an age-related factor(s) in blood that impacts neuroinflammation and cognitive deficits via effects on vascular cell adhesion molecule-1. Cognitive impairment was also linked to barrier dysfunction through activation of endothelial adenosine receptors (Adora2a) in a model of obesity and insulin-resistance [50].

The importance of understanding the mechanisms regulating BBB/NVU function is highlighted in a recent study on folate transport by Alam et al. [51]. Folate transport at the choroid plexus involves proton-coupled folate transport and folate receptor alpha and interference with such transport causes low CSF folate levels and neurodegeneration. Alam et al. [51] found that upregulating the reduced folate transporter at the BBB, by activating the vitamin D nuclear receptor with calcitriol, could compensate for the loss of choroid plexus transport.

Transporters at the cerebral endothelium play a critical role in BBB function. Two studies in 2019 highlighted how the plasma membrane distribution of transporters may be regulated. Zhang et al. [52] describes how contactin-associated protein 1 (CASPR1/CNTNAP1) binds to a subunit of Na^+^/K^+^-ATPase regulating trafficking to the plasma membrane. Similarly, Hoshi et al. [53] have identified the effects of ERM proteins (ezrin/radixin/moesin) on the location and activity of different transporters. ERM proteins may act as linkers between transporters and the cytoskeleton.

Although this section has focused on signaling and crosstalk within the NVU and cerebral endothelium, it should be noted that the BBB is also an endocrine tissue. It is a both a target for blood-borne hormones and a secretor of hormones into either the brain interstitial space or blood. These multiple roles of the BBB, as well as the effects of endocrine disease have been recently reviewed by Banks [54].

### Extracellular matrix

There is a growing awareness of the importance of the endothelial glycocalyx and basement membrane, and the brain extracellular matrix in BBB and NVU function in health and disease [55]. For example, there is recent evidence on the importance of the marked loss of the endothelial glycocalyx in cerebral malaria [56] where it was found that treating mice with cerebral malaria with either dexamethasone or antithrombin-3 could reduce glycocalyx loss, BBB disruption and mortality. Delsing et al. [57] also provided evidence of the importance of an extracellular matrix component, laminin, on astrocyte function, with iPSC-derived astrocytes having different gene expression profiles depending upon the type of laminin used. Yao and others have been dissecting the role of different basement membrane components on normal BBB physiology and how those components are affected by ischemic and hemorrhagic stroke or may contribute to stroke occurrence [58].

### Claudins at the cerebral endothelium

While the importance of claudin-5 as a tight junction protein at the cerebral endothelium is widely recognized [59], the potential role of other claudins [60] is more contentious. Uchida et al. [61] recently provided evidence in multiple sclerosis that claudin-11 is decreased at the BBB (and the blood-spinal cord and the blood-arachnoid barriers) contributing to barrier leakiness. In contrast, Castro Dias et al. [62, 63] found that loss of claudin-12 and claudin-3 had no effect on BBB permeability in mice and that claudin-3 was absent from the cerebral endothelium. Even the permeability at the choroid plexus, where claudin-3 is expressed, was unaffected by its deletion, although there may be a compensatory change in claudin-2 [63]. In a twist, Sladojevic et al. [64] found that in chronic stroke there is an upregulation of claudin-1 that destabilizes (rather than stabilizes) the BBB leading to long-term low-level barrier leakiness that affects functional recovery.

There is interest in the potential role of claudin-5 changes in psychiatric diseases [59]. For example, schizophrenia risk is greatly enhanced in patients with 22q11.2 deletion syndrome and the claudin-5 gene is within the deleted region. Previously, Greene et al. [65] found a claudin-5 variant, rs10314, is associated with reduced endothelial claudin-5 expression and the occurrence of that variant in the remaining 22q11.2 region increased the risk of schizophrenia. Recently, Guo et al. [66] reported this increased risk occurred in female, but not male patients.

## Choroid plexus and arachnoid membrane

### Choroid plexus

During the past few years, there has been emphasis on the importance of the choroid plexus as a site of leukocyte entry into brain in a variety of neurological conditions. Mottahedin et al. [67] have recently delineated the mechanisms induced by toll-like receptor-2 activation at the choroid plexus that result in leukocyte migration. Yin et al. [68] examined how apolipoprotein-E affects neuroinflammation and found it avidly binds a component of the classical complement cascade, C1q, at the choroid plexus inhibiting that cascade during inflammation. Apart from being a site of leukocyte migration between blood and CSF, the choroid plexus also has resident leukocytes (e.g. the epiplexus cells on the apical surface and cells in the stroma). There has been increased interest in the role of macrophages at all blood-CNS interfaces (perivascular, leptomeningeal, dural and choroid plexus) [69]. At the choroid plexus, epiplexus cell activation occurs in different hydrocephalus models [70, 71].

The choroid plexus epithelium displays a polarized distribution of transporters and channels involved in directional movement across the epithelium. For example, the polarity of the water channel, aquaporin-1, at the choroid plexus is reversed in obstructive hydrocephalus from the apical to the basolateral membrane [72] that might be a compensatory response. It will be important to know if there are similar changes in ion transport involved in CSF production.

Evidence suggests that inflammatory pathways at the choroid plexus regulate CSF secretion. Interestingly, Simpson et al. [73] showed that cytokines and other inflammatory mediators regulate a non-selective cation channel, transient receptor potential vanilloid-4 (TRPV4), at the epithelium. There is some controversy about the role of the apically located Na^+^/K^+^/Cl^−^-cotransporter (NKCC1) at the choroid plexus epithelium. NKCC1 can act as an influx or an efflux transporter dependent upon ion gradients. It has been proposed that it acts as an efflux transporter at the choroid plexus and is involved in CSF secretion but this has been questioned by Gregoriades et al. [74] who have provided evidence for influx activity. This is a difficult issue to resolve because different experimental preparations may alter cellular ion concentrations [75]. This is an important issue as NKCC1 is one potential target for altering CSF secretion and brain intracranial pressure [76]. It may be that NKCC1 may act as a sensitive regulator of CSF production with the ability to move ions into or out of CSF.

### Arachnoid membrane and dura mater

The description of lymphatic vessels within the dura mater (see below) has led to an upswing in interest in the meninges. There has also been specific interest in the arachnoid membrane, one site of the blood-CSF barrier, where the absolute protein amount of a variety of transporters (e.g. p-glycoprotein and organic anion transporters) is greater than at the choroid plexus in pig and transporter distribution is polarized (e.g. CSF- or blood-facing) [77]. There are also continued efforts to describe the anatomy and immunology of the cerebrovasculature [78] and to understand the meninges as an immune-blood–brain interface [79].

## Cerebrospinal fluid and the glymphatic hypothesis

### CSF and glymphatic dynamics

There continues to be a lively debate about the proposed glymphatic system (e.g. [80, 81] and review [82]) and about CSF dynamics (e.g. [83, 84]). 2019 has seen many studies addressing aspects of the glymphatic system, para-arterial entry from CSF to brain, fluid flow within brain, where aquaporin 4 is proposed to play a critical role, and paravenous efflux from brain to CSF. MRI is now being used to study different elements of this system [85–91]. Paravenous clearance of solutes from brain has been less well studied than entry. Van Veluw et al. [92] have recently provided evidence on the importance of vasomotion in the clearance of solutes from brain in awake mice. New tools for studying the glymphatic system are welcome and Munk et al. [93] have recently identified platelet-derived growth factor-B as being essential for glymphatic development.

There continues to be great interest in the role of meningeal lymph vessels in CSF drainage. Ahn et al. [94] have now described meningeal lymph vessels at the base of the brain that have lymphatic valves (in contrast to the dorsal meningeal lymph vessels) and are involved in clearing macromolecules from CSF. Aging impacts those vessels potentially contributing to age-related neurological conditions. However, here are other routes for CSF egress to the lymphatic system. It has long been known that the cribriform plate is a major exit route, as further delineated by Norwood et al. [95], while Hsu et al. [96] have described how neuroinflammation impacts that route, changes that differ from meningeal lymph vessels.

A potential implication of a rapid paravascular entry of CSF from the subarachnoid space into brain relates to drug delivery, i.e. CSF delivered agents may rapidly reach targets within brain parenchyma. However, Ma et al. [97] found in conscious animals a rapid clearance of CSF-infused tracers to the lymph system significantly limits potential brain penetration.

There has been debate over the relative importance of choroid plexus and extrachoroidal sources of CSF. The choroid plexus expresses the water channel aquaporin-1, while ependyma and the glia limitans (as well as astrocyte endfeet) express aquaporin-4. Trillo-Contreras et al. [98] examined CSF outflow, intraventricular pressure and ventricular volume in aquaporin-1 and -4 knockout mice and the double knockout and concluded both are important in CSF production.

There has been much interest in the effects of the sleep/wake cycle on the clearance of metabolic waste products from the brain, particularly potentially toxic metabolites including β-amyloid (Aβ) and tau and this was reviewed in-depth by Hladky and Barrand [99]. Recently, Holth et al. [100] have examined the effects of the sleep/wake cycle on brain interstitial fluid and CSF tau and found high levels during wakefulness and sleep deprivation. Hablitz et al. [101] found that glymphatic influx (movement of tracers from CSF to brain) correlates with cortical delta power of electroencephalograms (EEG) and negatively with beta power and heart rate. The complexity of the changes in the fluid compartments during sleep/wakefulness in humans is highlighted by a study by Demiral et al. [87] using measurements of the apparent diffusion coefficient (ADC) for water with MRI. They found different effects of sleep within the brain depending on brain region and changes in CSF volume.

In another twist, there is also evidence of circadian rhythms in gene expression at the BBB and barrier function (reviewed in [102]). At the inner blood-retina barrier, Hudson et al. [103] have found that claudin-5 gene expression is regulated by BMAL-1, a clock gene. In 2018, there was also work on the importance of the choroid plexus in the circadian clock [104]. It is likely that the effects of the sleep cycle and circadian rhythms on barrier function and brain fluid movement will involve multiple sites. There has now been a study of the impact of long-duration space flight on ventricular CSF volume [105]. There was a ~ 12% increase in volume in each of the ventricles post-flight compared to pre-flight, perhaps due to reduced CSF resorption. While the magnitude of the increase was reduced 7-months post-flight, there was still a significant increase over pre-flight status (e.g. 7.7% increase in lateral ventricle volume).

### Communication via CSF

Kaiser et al. [106] have provided important evidence for the role of choroid plexus-produced WNT5a in regulating hindbrain development. The hydrophobic WNT5a is transported through the CSF system bound to lipoprotein particles rather than in exosomes. There is also new evidence that choroid plexus-produced microRNA-204 regulates neural stem cells in the subependymal zone of the adult brain [107] and that OTX2, a homeoprotein transcription factor secreted by choroid plexus, regulates adult neurogenesis [108]. Esposito et al. [109] recently examined the role of brain to cervical lymph node communication in inflammation after stroke and showed that blocking such signaling reduces the peripheral inflammatory response and brain injury.

### CSF biomarkers

CSF biomarkers continue to be investigated for potential diagnosis of various neurological conditions including different forms of neurodegeneration [110–113]. In Alzheimer’s disease (AD), many studies have focused on increases in CSF Aβ or tau species, proteins that are harmful, or injury markers such as neurofilament light chain. However, Ewers et al. [114] examined whether increases in CSF soluble TREM2 in patients might be associated with protection against AD. They found soluble TREM2 was associated with less cognitive and clinical decline in AD patients. Differences in protective molecules may contribute to interpatient variations in outcome as well as potentially identifying new therapeutic targets. Other important examples of the use of CSF biomarkers in 2019 include Blennow et al. [115], who found that a specific tau fragment in CSF correlates well with tau imaging on positron emission tomography (PET). Also, Kuiperij et al. [116] found that CSF apolipoprotein D levels can distinguish AD patients with and without cerebral amyloid angiopathy as well as from control patients. Similarly, Jeppsson et al. [117] found that idiopathic normal pressure hydrocephalus (iNPH) patients could be distinguished from patients with cognitive and movement disorders by having a lower combination of CSF markers: total-tau, Aβ40 and monocyte chemoattractant protein-1. A concern with CSF biomarker studies is potential effects of blood contamination during sampling and Batllori et al. [118], examining CSF metabolic biomarkers, found that CSF centrifugation prior to freezing for bio-banking minimized the effects of blood contamination for many biomarkers.

While most attention has focused on CSF proteins or metabolic markers [118], another recent approach is CSF DNA analysis. Thus, tumor DNA in CSF may help identify tumor mutations without the need for biopsies. Miller et al. [119] found that ~ 50% of glioma patients had tumor DNA in lumbar CSF samples with a variety of mutations. This approach shows great promise for enabling changes in tumor genetic makeup to be tracked with time or treatment.

## The brain barriers and brain fluids in disease

### Hydrocephalus

To date, inheritable congenital hydrocephalus has been linked to eight genes. One gene is MPDZ that encodes a scaffolding protein and Yang et al. [120] investigated the underlying cause of the hydrocephalus in Mpdz knockout mice finding that the loss of the gene caused choroid plexus hyperpermeability. This may be due to increased transcytosis but they also reported a loss of tight junction proteins, ZO-1 and JAM-C, and an increased Na^+^/K^+^/Cl^−^ cotransporter (NKCC1) expression at the choroid plexus. Recently, Diets et al. [121] identified a pathogenic variant of SMARCB1, which encodes a protein involved in chromatin remodeling. Four patients with this mutation had choroid plexus hyperplasia and resultant hydrocephalus and severe intellectual disability. Another recently identified human mutation associated with hydrocephalus is in the K^+^/Cl^−^-cotransporter KCC3 (SLC12A) [122]. How the mutation leads to hydrocephalus is still unclear (e.g. which cell type(s) is involved).

### Intraventricular post-hemorrhagic hydrocephalus (IVH)

Intraventricular hemorrhage commonly results in hydrocephalus in low birth weight infants and results in serious neurological problems [123]. Research has been directed at mitigating these effects: for example, Romantsik et al. [124] found that α1-microglobin, a heme and free radical scavenger, protects against periventricular damage in a preterm rabbit model of intraventricular hemorrhage. Ding et al. [125] have found evidence of an impaired glymphatic system in a model of germinal matrix hemorrhage and that olomoucine, an inhibitor of astrogliosis, ameliorated that impairment, reduced hydrocephalus and improved neurological outcome. Fast diffusion MR imaging is helping to identify abnormal periventricular tissue in premature infants with post hemorrhagic hydrocephalus [126].

### Idiopathic normal pressure hydrocephalus (iNPH)

Diagnosing idiopathic normal pressure hydrocephalus (iNPH), a condition that affects the elderly, poses many challenges and identifying patients for surgery is difficult. However, neuroimaging techniques are becoming more sophisticated for non-invasive diagnosis and the development of guidelines (reviewed in [127]). Co-morbidities may impact the condition: for example, Hudson et al. [128] recently reviewed the occurrence of diabetes mellitus in patients with iNPH. They found much higher incidence (~ 2-4 fold) in age- and cohort-matched patients with iNPH. This may further impact these patients and an elucidation of underlying mechanisms is needed. A transcranial magnetic stimulation study [129] in iNPH patients has shown impaired cholinergic transmission in NPH patients, which could contribute to gait disturbance and neurological defects. As noted above, there has been increased interest in the impact of long non-coding RNAs in neurological conditions and Shi et al. [130] identified 1575 changes in the expression of such RNAs in a kaolin model of hydrocephalus in mice. They particularly regulate inflammatory pathways.

There is a suggestion that the glymphatic system may be involved in hydrocephalus pathogenesis since a defective system might impair fluid flow, reduce removal of toxic products and contribute to neurological deficits. Impairments in glymphatic circulation have been reported in iNPH patients [88], and also in spontaneously hypertensive rats that begin to develop hydrocephalus several weeks after birth [91]. MR diffusion imaging studying paravascular fluid flow in patients and controls, supports the concept of an impaired flow system and such imaging could be used to aid diagnosis for patients with iNPH [131]. Aquaporin 4 is thought to play a key role in the glymphatic system and Hasan-Olive et al. [89] found a reduced density of aquaporin 4 in astrocytic endfeet along cortical microvessels in iNPH patients. That group also found evidence of pathological mitochondria in the perivascular astrocytic endfeet in those patients [132].

Cilia defects have been implicated in hydrocephalus pathology for some time and primary ciliopathies affect various aspects of brain development, including hydrocephalus [133]. For example, Morimoto et al. [134] identified a loss of function mutation in CFAP3, a cilia-associated protein, in a Japanese family where some members have NPH. In addition, they found that Cfap3-deficient mice also develop hydrocephalus. Similarly, Chiani et al. [135] found that loss of Ccdc151, which causes a primary ciliary dyskinesia, is associated with hydrocephalus in mice. The paucity of known mechanisms for iNPH highlights a desperate need for new therapeutic targets and approaches for hydrocephalus and associated neural injury.

### Idiopathic intracranial hypertension (IIH)

The underlying cause of idiopathic intracranial hypertension (IIH) is uncertain: it primarily affects obese women of reproductive age, is of increasing incidence and can lead to vision loss [136]. A diagnostic feature is the presence of increased peri-optic CSF determined by neuroimaging [137]. O’Reilly et al. [138] examined the systemic and CSF androgen metabolome in patients with IIH and found a unique signature of androgen excess and provide evidence that this may affect CSF secretion. There is also evidence that these patients may have defects in CSF drainage due to increased sagittal sinus venous pressure [139] and that there may be BBB dysfunction as measured by extravasated fibrinogen in the brain [136].

### Meningitis

There have been several interesting papers on meningitis this past year. Ma et al. [140] found that *streptococcus* expresses a protein containing a Fic domain that disrupts the BBB by activating moesin allowing bacterial entry into the brain. In another study, Kim et al. [141] found that *streptococcus* exposure inhibited p-glycoprotein in brain endothelial cells and this effect was replicated in an in vivo meningitis model. This may impact drug regimen design. Mohanty et al. [142] identified a novel potential target for pneumococcal meningitis. In infections, neutrophils release neutrophil extracellular traps (NETs) that snare and kill bacteria. However, while NETs are produced in CSF during pneumococcal meningitis, it appears that they have a detrimental effect preventing bacterial clearance.

### The contribution of brain vascular dysfunction to different disease states

A vast array of neurological events and conditions (if not all) impact the BBB and the NVU. For example, different degrees of traumatic brain injury cause BBB dysfunction. Thus, there is evidence of BBB dysfunction in adolescent rugby players to professional mixed martial arts fighters [143] and Yoo et al. [144] found that BBB dysfunction after mild traumatic brain injury was associated with post-concussion syndrome (events such as headaches and dizziness lasting for weeks). In more severe traumatic brain injury, there is evidence that cerebral microvascular injury is a therapeutic target by contributing to neurodegeneration [145]. The recent CRASH-3 clinical trial [146], using tranexamic acid to limit cerebral bleeding after traumatic brain injury, significantly improved mortality.

There is a growing understanding of the importance of BBB dysfunction in multiple forms of acute brain injury and chronic neurodegeneration [147]. Sweeney et al. [148] have recently reviewed the importance of vascular dysfunction in AD. Interestingly, Nation et al. [149] also found that patients developing early cognitive dysfunction have evidence of BBB dysfunction even without increased Aβ or Tau. Milikovsky et al. [150] found evidence that BBB dysfunction underlies electroencephalogram changes in AD in patients and animal models. One marker of cerebrovascular dysfunction in the elderly is the presence of cerebral microbleeds and in a meta-analysis, Debette et al. [151] reported that patients with such microbleeds on MRI had 1.9- and 3.8-fold increased risk of ischemic and hemorrhagic stroke.

Much focus on BBB changes in disease states has centered on inflammatory changes and increased permeability, either due to endothelial tight junction disruption, increased transcytosis or even endothelial cell death (for a recent review of the latter see [152]). However, disease states affect the BBB and the NVU at many levels. For example, there are changes in BBB transport in stroke, AD and psychiatric disorders [153–155]. Krueger et al. [156] recently highlighted the importance of endothelial edema after stroke. Because of the prevalence of vascular changes across neurological disorders, there have been studies looking for commonalities (and differences). Two recent studies examining the transcriptome are by Munji et al. [157] on the brain endothelial response and Guo et al. [158] on the vasculome in brain and heart.

## Drug delivery

Entry of drugs across the BBB remains a major hurdle for developing therapies for neurological disorders. In addition, the distribution of therapeutics entering the brain may be inhomogeneous, raising difficulties in ensuring proper target engagement. Vendel et al. [159] reviewed the complexity of modelling brain distribution for therapeutics and concluded that more work is still needed. Another example of complexity of drug delivery to brain is the intranasal route. Lochhead and Davis [160] have recently reviewed how the perineural and perivascular pathways are important in such delivery. Enhanced drug delivery across the BBB can be achieved by conjugating drugs to antibodies targeting receptors involved in transcytosis. Much of such work has focused on targeting the BBB transferrin receptor and Johnsen et al. [161] provide a review of that work. There has also been a thrust for using antibody fragments rather than full-length antibodies and Belanger et al. [162] reviewed current work on the use of small single-domain antibodies. Alternatively, Thom et al. [163] used a peptide from melanotransferrin to enhance delivery of an interleukin-1 receptor antagonist to reduce neuropathic pain in mice. Similarly, Wu et al. [164] used a bacteriophage-derived peptide to target the transferrin receptor and the receptor for advanced glycation-end products (RAGE). They complexed the peptide with a siRNA to down-regulate β-secretase within the brain.

There has been great interest in using variants of adeno-associated virus (AAV) serotypes that can cross the BBB for gene therapy delivery. An example of this approach has been the neurotropic AAV-PHP.B variant. Hordeaux et al. [165] have identified that this variant enters the mouse brain by binding to a protein, LY6A but they also note that primates have no homolog of LY6A which may explain why AAV-PHP.B does not increase CNS transduction in non-human primates. There is a need for more information on differences in BBB protein expression across species. In addition to gene delivery, the AAV technology can be applied to mechanistic studies. For example, the role of ferroportin in iron import to the brain and retina was demonstrated by deletion of this protein from endothelial cells of the brain and retina [166].

Some drugs may be substrates for solute transporters at the BBB. For example, Albekairi et al. [153] have found that the potent opioid receptor agonist, biphalin, is a substrate of the organic anion transporting polypeptide OATP1. The expression of BBB solute transporters can vary in neurological conditions giving potential opportunities for drug delivery: for example, OATP1 is increased in stroke [153].

Focused ultrasound has been used to transiently open the BBB to enhance drug delivery in vivo in animal models [167, 168] and, as discussed below, in the clinic [169, 170]. An interesting further use of ultrasound-induced barrier disruption has been to allow entry of a contrast agent into the brain of patients which can then be tracked non-invasively by MRI to examine clearance routes (e.g. the glymphatic system) [171]. Another approach to modulate the BBB has been to target brain endothelial junction proteins and a series of peptides have been developed that bind to claudins [172]. In 2019, one of those peptides was used to down-regulate claudin-1 at the BBB long-term after stroke as that particular claudin causes barrier instability [64]. Similarly, Yang et al. [173] have used a peptide targeting E-cadherin to enhance brain uptake of eflornithine, a drug used to treat trypanosomiasis.

There has been a wealth of studies in 2019 on potential therapeutics for neurological disorders where changes at the BBB have been an endpoint. It is usually unclear whether the therapeutic is having a direct effect on the brain endothelium or a secondary effect via neuroprotection. This is an important point as dosing regimens will differ greatly for an endothelial target versus a target beyond the BBB. There continues to be a need for more studies that address the specific role of endothelial dysfunction in neurological disorders.

## Translation to the clinic

An ultimate goal of preclinical research is to translate data to the clinic and 2019 has provided several examples of progress on that front. Reperfusion (induced by tissue plasminogen activator or thrombectomy) is the only current therapy for ischemic stroke, but reperfusion can lead to cerebral hemorrhage. There has, therefore, been great interest in targeting the cerebral endothelium to prevent such hemorrhage; i.e. combining neuro- and endothelial protectants [174]. Preclinically, a recombinant variant of activated protein C is endothelial protective and a Phase II clinical trial (RHAPSODY) using that drug in combination with reperfusion has just been completed with promising results [175].

Brain drug delivery remains a major challenge for a wide range of neurological disorders. Ultrasound can be used to induce transient BBB disruption to enhance drug delivery. For example, Idbaih et al. [170] have just reported results on an ultrasound device implanted in the skull of patients with recurrent glioblastoma. They used the low-intensity pulsed ultrasound to disrupt the BBB repeatedly during sequential carboplatin treatments. That preliminary study on a limited number of patients showed the safety of the device and procedure and had a tendency towards improving survival. Similarly, Abrahao et al. [169] recently showed the safety of MR guided focused ultrasound in patients with amyotrophic lateral sclerosis (ALS).

The choroid plexus is a source of neurotrophins and a formulation consisting of encapsulated pig choroid plexus cells has been developed that can be implanted into patients. This underwent a phase IIb clinical trial in patients with Parkinson’s disease (at least 5 years after onset). While the formulation was safe, it did not improve outcomes in those patients [176]. One drug formulation that is being pursued to treat brain edema in multiple neurological conditions is an intravenous preparation of glibenclamide (BIIB093). That includes clinical trials in brain ischemia and traumatic brain injury [177]. Glibenclamide targets SUR1-TRPM4 channels that are upregulated in several NVU cell types after injury. Brain edema treatments have not changed for decades and a new drug would be very welcome.

## In memoriam

Brain barriers and brain fluid research sadly lost two major figures in 2019: Malcolm Segal and Ed Stopa.

### Malcolm B. Segal (August 1st, 1937–July 29th, 2019)

Malcolm Segal spent many years in the Division of Physiology at United Medical and Dental School of Guy’s and St. Thomas’ Hospitals, where he rose to Chairman. After the merger with King’s College, he continued in the Department of Physiology until his retirement. During his career, Malcolm made many seminal findings on choroid plexus and CSF and helped develop the ventriculo-cisternal perfusion technique, as well as methods for determining the resistance to CSF drainage and establishing the isolated perfused sheep choroid plexus preparation. His studies covered a wide range from CSF secretion and drainage [178, 179], choroid plexus transport of many compounds [180–183] and the impact of development and aging on the blood–brain barriers [184, 185]. Malcolm also co-authored with Hugh Davson and Keasley Welch the essential tome on all things CSF, *The Physiology and Pathology of the Cerebrospinal Fluid* [186] and subsequently *Physiology of the CSF and Blood*–*brain Barriers* with Hugh Davson [187]. A reflection on the important role of Malcolm had in our understanding of choroid plexus/CSF physiology and in the training of many current scientists has recently been published in *Fluids and Barriers of the CNS* [188]. He will be greatly missed.

### Edward G. Stopa (July 6th, 1954–September 18th, 2019)

Ed Stopa was Professor of Pathology and Neurosurgery at Brown University as well as Director of Neuropathology at Rhode Island Hospital. While Ed was best known for his work on AD and other forms of dementia [189–192], he also worked on hydrocephalus [193, 194] and contributed to our understanding of the roles of CSF [195, 196] and the BBB [192, 197]. He served as an Editorial Board member of *Fluids and Barriers of the CNS*. The field can ill-afford to lose a trained neuropathologist with his range of interests and expertise. An obituary can be found in *Journal of Alzheimer’s Disease* [198].

## Conclusions

We would like to thank the readers, authors, reviewers and editorial board members of *Fluids and Barriers of the CNS* for their support in 2019. As shown by these ‘highlights of 2019’, the field is vibrant with new ideas and important findings. May that continue in 2020!

## Data Availability

Not applicable.
